# CAR T-cell vs bispecific antibodies in relapsed/refractory mantle cell lymphoma: a systematic review and meta-analysis

**DOI:** 10.3389/fonc.2026.1860307

**Published:** 2026-08-11

**Authors:** Meng-jiao Li, Ling Qiu, Dan Chen, Cai-yang Li, Ying He, Yan-ling Li, Ya-li Cen, Shi-hui Ren, Hao Yao, Fang-yi Fan

**Affiliations:** 1 Department of Hematology, The General Hospital of Western Theater Command, Chengdu, Sichuan, China; 2 Department of Clinical Medicine, North Sichuan Medical College, Nanchong, Sichuan, China; 3 Institute of Basic Medicine, North Sichuan Medical College, Nanchong, Sichuan, China; 4 College of Medicine, Southwest Jiaotong University, Chengdu, Sichuan, China; 5 Reproductive Medicine Center, The General Hospital of Western Theater Command, Chengdu, Sichuan, China; 6 Tissue Stress Injury and Functional Repair Key Laboratory of Sichuan Province, Chengdu, Sichuan, China

**Keywords:** bispecific antibodies, CAR T-cell therapy, mantle cell lymphoma, meta-analysis, relapsed/refractory

## Abstract

**Background:**

Mantle cell lymphoma (MCL) is an aggressive B-cell lymphoma with limited treatment options for relapsed/refractory (R/R) cases. CAR T-cell therapy and CD20×CD3 bispecific antibodies are promising immunotherapies, but no study has systematically compared their efficacy and safety in R/R MCL.

**Methods:**

This systematic review and meta-analysis was conducted in accordance with the PRISMA 2020 guidelines and registered in PROSPERO (CRD42024622564). A comprehensive search of PubMed, Embase, and the Cochrane Library (from inception to October 27, 2024) was performed to identify studies evaluating CAR T-cell therapy and bispecific antibodies in R/R MCL (third-line or later). Random-effects and fixed-effects models were used to calculate pooled complete response (CR) rates, overall response (OR) rates, and Grade ≥3 adverse events. Sensitivity analyses were conducted to assess the robustness of the results.

**Results:**

Five studies (237 patients) were included. Pooled CR rates were 0.63(95% CI, 0.51-0.74)for bispecific antibodies and 0.70(95%CI, 0.63-0.77) for CAR T-cell therapy (P <0.05). OR rates were similar (0.75 vs. 0.88). Grade ≥3 cytokine release syndrome rates were comparable (0.06 vs. 0.06), but neurotoxicity cannot be compared.

**Conclusion:**

CAR T-cell therapy demonstrated higher CR rates versus bispecific antibodies, supporting their role as effective third-line treatments for R/R MCL. Randomized trials are needed to confirm these findings.

**Systematic Review Registration:**

https://www.crd.york.ac.uk/prospero/, identifier CRD42024622564.

## 1 Introduction

Mantle cell lymphoma (MCL) is a distinct subtype of lymphoma characterized by significant clinical heterogeneity. High-risk biological features, including blastoid morphology, high cell proliferation (Ki-67 > 30%), and TP53 mutations, have been identified as factors associated with poor prognosis (1, 2). While chemotherapy remains the frontline treatment for MCL, Bruton’s tyrosine kinase inhibitors (BTKi) are the preferred targeted therapies, particularly in cases of early relapse (3). For high-risk patients eligible for transplantation, allogeneic hematopoietic stem cell transplantation (allo-HCT) is another effective option, achieving long-term disease-free survival in approximately 30% of cases (4). However, a significant number of patients demonstrate suboptimal responses to these treatments, particularly those with disease progression under BTKi therapy, who often exhibit highly aggressive clinical features and poor outcomes (5).

Recent advances in immunotherapy have brought hope to patients with relapsed or refractory (R/R) MCL, especially with the emergence of novel therapies such as chimeric antigen receptor (CAR) T-cell therapy and CD20×CD3 bispecific antibodies. Both approaches leverage T-cell-mediated mechanisms to target tumor cells, representing a paradigm shift in the treatment of R/R MCL. CAR T-cell therapy involves engineering patient-derived T cells to recognize tumor antigens and elicit robust anti-tumor responses. Among the CAR T-cell therapies, brexucabtagene autoleucel, targeting CD19, has demonstrated the most significant efficacy for MCL and is associated with durable responses even in high-risk populations (6, 7). Conversely, bispecific antibodies, which simultaneously bind to tumor antigens and T-cell receptors, offer a readily available off-the-shelf treatment option with distinct advantages, including direct T-cell-mediated tumor cell killing, high affinity, and potentially lower production costs (8).

Since 2017, the U.S. Food and Drug Administration (FDA) has approved several CAR T-cell therapies and bispecific antibodies for hematologic malignancies, including MCL. However, direct comparative studies evaluating the efficacy and safety of these two T-cell-mediated therapies in R/R MCL are scarce, posing challenges for clinical decision-making. Given the difficulty of conducting head-to-head randomized trials, a meta-analysis synthesizing existing clinical data offers a valuable approach to assess their relative effectiveness and safety.

This study aims to perform a systematic meta-analysis comparing the efficacy and safety profiles of CAR T-cell therapies and bispecific antibodies in the third-line or later treatment of R/R MCL. By evaluating endpoints such as complete response (CR) rates, overall response (OR) rates, and the incidence of severe adverse events, this analysis seeks to provide clinicians with critical insights to guide therapeutic decisions for this challenging disease.

## 2 Methods

### 2.1 Ethical statement

This meta-analysis was conducted using previously published data and did not involve any new research involving human participants or animals. All included studies had obtained ethical approval from their respective institutional review boards as reported in the original publications. As this analysis utilized publicly available data, additional ethical approval was not required.

### 2.2 Literature search

This systematic review and meta-analysis was conducted in accordance with the PRISMA 2020 guidelines and prospectively registered in the International Prospective Register of Systematic Reviews (PROSPERO; registration number: CRD42024622564). A systematic search of PubMed, Embase, and the Cochrane Library databases was performed to identify studies evaluating the efficacy and safety of CAR T-cell therapy or CD20×CD3 bispecific antibodies in relapsed/refractory mantle cell lymphoma (R/R MCL). The search strategy included the keywords “mantle cell lymphoma,” “chimeric antigen receptor,” and “bispecific antibody,” along with their related synonyms and derivatives. The search period was from the establishment of each database to October 27, 2024. Additionally, conference abstracts and unpublished studies were manually screened to ensure comprehensive coverage. All retrieved records were independently reviewed for eligibility based on the predefined inclusion and exclusion criteria.

### 2.3 Inclusion and exclusion criteria

Studies were included if they met the following criteria:

Studies were included if they met the following criteria: Prospective interventional clinical trials or large real-world cohort studies evaluating CD20×CD3 bispecific monoclonal antibodies or CAR T-cell therapy for R/R MCL. Studies reporting outcomes with clearly defined therapeutic doses. Studies reporting at least one efficacy endpoint, including complete response (CR) rate or overall response (OR) rate, and safety endpoints such as Grade ≥3 cytokine release syndrome (CRS) or neurological adverse events. Two reviewers (M.J.L. and L.Q.) independently screened the titles, abstracts, and full texts for eligibility. Disagreements were resolved by consensus or by involving a third reviewer (D.C.).

Exclusion criteria included:

Preclinical studies, case reports, reviews, or articles unrelated to the research topic.

Studies with ambiguous or incomplete endpoints or unclear treatment doses. Studies focused on lymphoma subtypes other than MCL. Duplicated publications or studies with insufficient data for analysis. Studies assessing combination therapies or radiotherapy as the primary intervention.

### 2.4 Data extraction and risk of bias assessment

Two independent reviewers extracted data from the eligible studies, including first author, year of publication, study design, sample size, patient characteristics (e.g., median age, number of prior therapy lines, proportion of stage III/IV patients, Ki67 ≥30%, BTKi exposure, and TP53 mutation status), therapeutic regimens, and clinical outcomes. Efficacy outcomes included CR and OR rates, while safety endpoints encompassed the incidence of Grade ≥3 CRS and neurological toxicities. Any discrepancies in data extraction were resolved through discussion or consultation with a third reviewer.

The quality of included studies was assessed using the Methodological Index for Non-Randomized Studies (MINORS), with a maximum score of 16 for non-randomized studies and 24 for randomized trials.

### 2.5 Statistical analysis

The primary endpoint of this analysis was the pooled CR rate. Secondary endpoints included pooled OR rates and the incidence of Grade ≥3 adverse events, including CRS and neurotoxicity. Meta-analytical methods were employed using both random-effects and fixed-effects models to calculate pooled effect sizes and their corresponding 95% confidence intervals (CI). The degree of heterogeneity was assessed using the I^2^ statistic, with values >50% indicating substantial heterogeneity. Based on the degree of heterogeneity, the appropriate model was selected for pooled analysis.

Sensitivity analyses were performed to assess the robustness of the results, including: Comparison of results between random-effects and fixed-effects models. Leave-one-out analyses to evaluate the influence of individual studies on the pooled estimates.

All statistical analyses were performed using R Studio. A P-value of <0.05 was considered statistically significant, and results were visualized using forest plots and I^2^ statistics to represent pooled estimates and heterogeneity.

## 3 Results

### 3.1 Literature search

**Figure 1:**
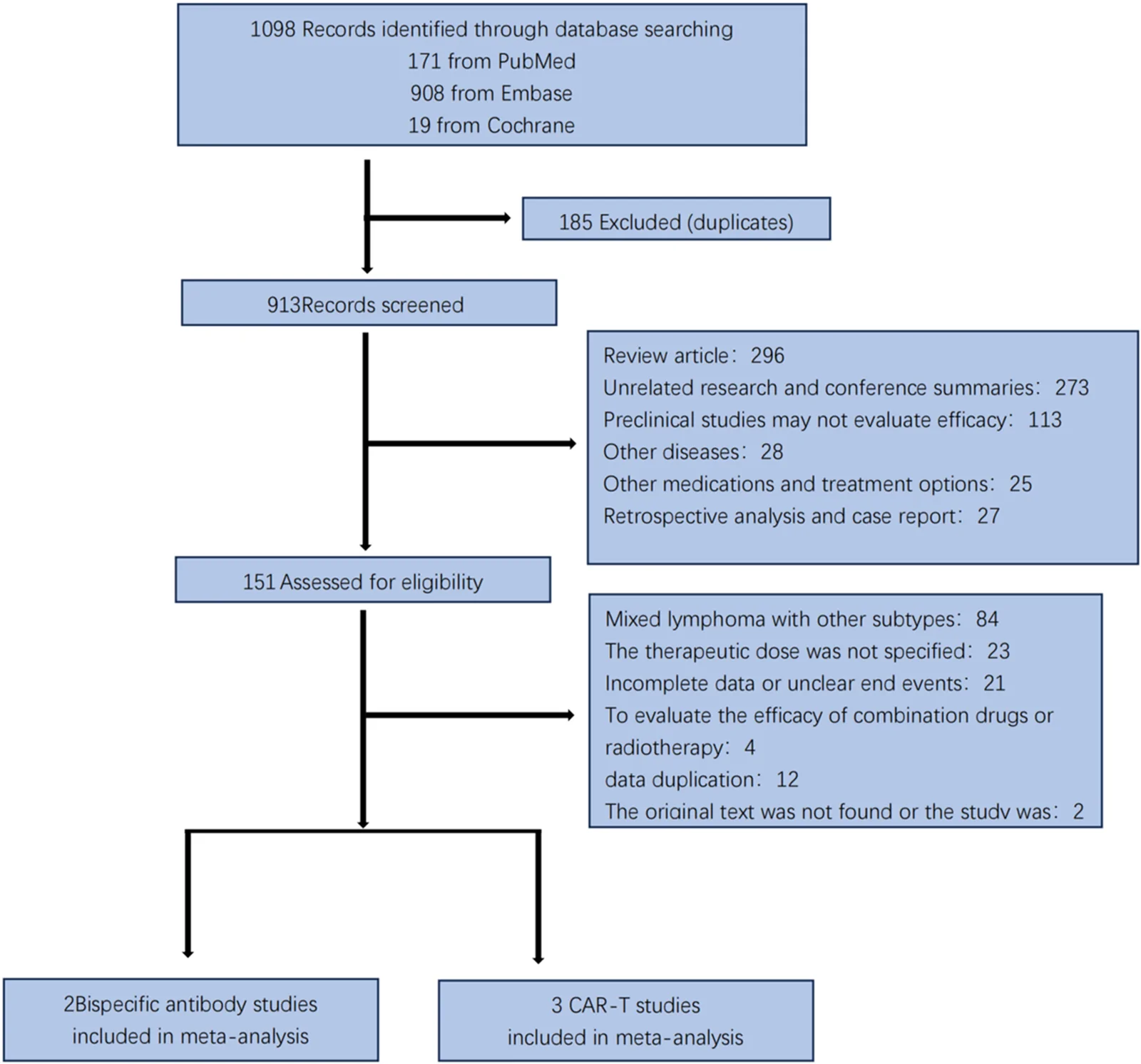
Trial selection flow diagram. Flow diagram illustrating the process of study selection for the meta-analysis. A total of 1098 records were identified through database searching, including 171 from PubMed, 908 from Embase, and 19 from Cochrane. After excluding 185 duplicates, 913 records were screened. From these, 151 articles were assessed for eligibility, while the remainder were excluded due to reasons such as review articles, unrelated research, preclinical studies, other diseases, or retrospective analysis. After detailed review, 2 studies on bispecific antibodies and 3 studies on CAR-T therapies were included in the final meta-analysis.

A total of 1098 studies were initially retrieved from PubMed, Embase, and the Cochrane Library databases. After removing 185 duplicates, 913 records were screened. Based on title and abstract review, 762 studies were excluded for being unrelated to the research topic. The full text of 151 studies was assessed, and 139 studies were further excluded for reasons such as lack of relevant outcomes, unclear dosing, or non-MCL populations. Ultimately, 5 studies were included in the meta-analysis (9–13), comprising 2 studies on bispecific antibodies (9, 10) and 3 studies on CAR T-cell therapies (11–13). These studies included 237 patients in total (**Figure 1**).

### 3.2 Study characteristics

The 5 included studies were published between 2021 and 2024. Baseline characteristics of the studies are summarized in **Tables 1**, **2**. The 2 bispecific antibody studies evaluated glofitamab (9) (n=1) and mosunetuzumab (10) (n=1), while the 3 CAR T-cell studies assessed KTE-X19 (11) (n=1), liso-cel (12) (n=1) and brexu-cel (13) (n=1). The median number of prior therapies ranged from 2 to 3 across the included studies. Quality assessment using the Methodological Index for Non-Randomized Studies (MINORS) indicated scores ranging from 11 to 14, reflecting moderate-to-high study quality (**Table 3**).

**Table 1:** Baseline characteristics of included studies.

Serial number	ClinicalTrials.gov identifier	Regimen	No	Median age	Median no. of previous therapy	Prior BTKi, %	Ki67≥30, %
1.	NCT03671018	Mosunetuzumab	20	68	3	100	70
2.	NCT03075696	Glofitamab	60	—	2	51.7	—
3.	—	Brexu-cel	19	67	2	100	85
4.	NCT02631044	Liso-cel	88	68.5	3	94	75
5.	NCT02601313	KTE-X19	68	65	3	100	82

Percentages are based on the evaluable patient population for whom the specific characteristic was reported in the original study. Data for some parameters were not available for all studies (e.g., ‘—’ indicates data not reported).

Summary of baseline characteristics from included studies, including regimen, number of patients, median age, median number of prior therapies, proportion of patients with prior BTKi therapy, and percentage of patients with Ki67 ≥30%.

**Table 2:** Additional patient and disease characteristics.

Serial number	Prior ASCT, %	MIPI≥6 %	TP53 %	median follow-up	Stage III/IV, %	Prior CAR T, %
1.	—	40	20	7.2	95	35
2.	—	—	—	17.7	—	—
3.	43	63	20	5	84	—
4.	33	9	23	16.1	—	—
5.	43	56(≥4)	17	35.6	—	—

Summary of additional patient and disease characteristics from included studies, including percentage of patients with prior ASCT, MIPI ≥6, TP53 mutation, median follow-up duration (in months), and proportion of patients in Stage III/IV disease.

**Table 3:** Quality assessment of the included studies.

Study	D1	D2	D3	D4	D5	D6	D7	D8	T
Michael L. Wang, MD2023	2	2	2	2	0	2	2	0	12
Tycel Phillips MD2024	2	1	2	2	0	2	2	0	11
Gloria Iacoboni2021	2	2	2	2	0	2	2	0	12
Michael Wang, MD2023	2	2	2	2	0	2	2	2	14
Michael Wang, MD2022	2	0	2	2	0	2	2	2	12

D1: Clear research objectives; D2: Consistency of patients; D3: Data collection; D4: Appropriate end-point indicators reflecting the objectives; D5: Objectivity of end-point indicators; D6: Sufficient follow-up time; D7: Loss to follow-up rate < 5%; D8: Estimation of sample size.

### 3.3 Pooled efficacy outcomes

**Figure 2:**
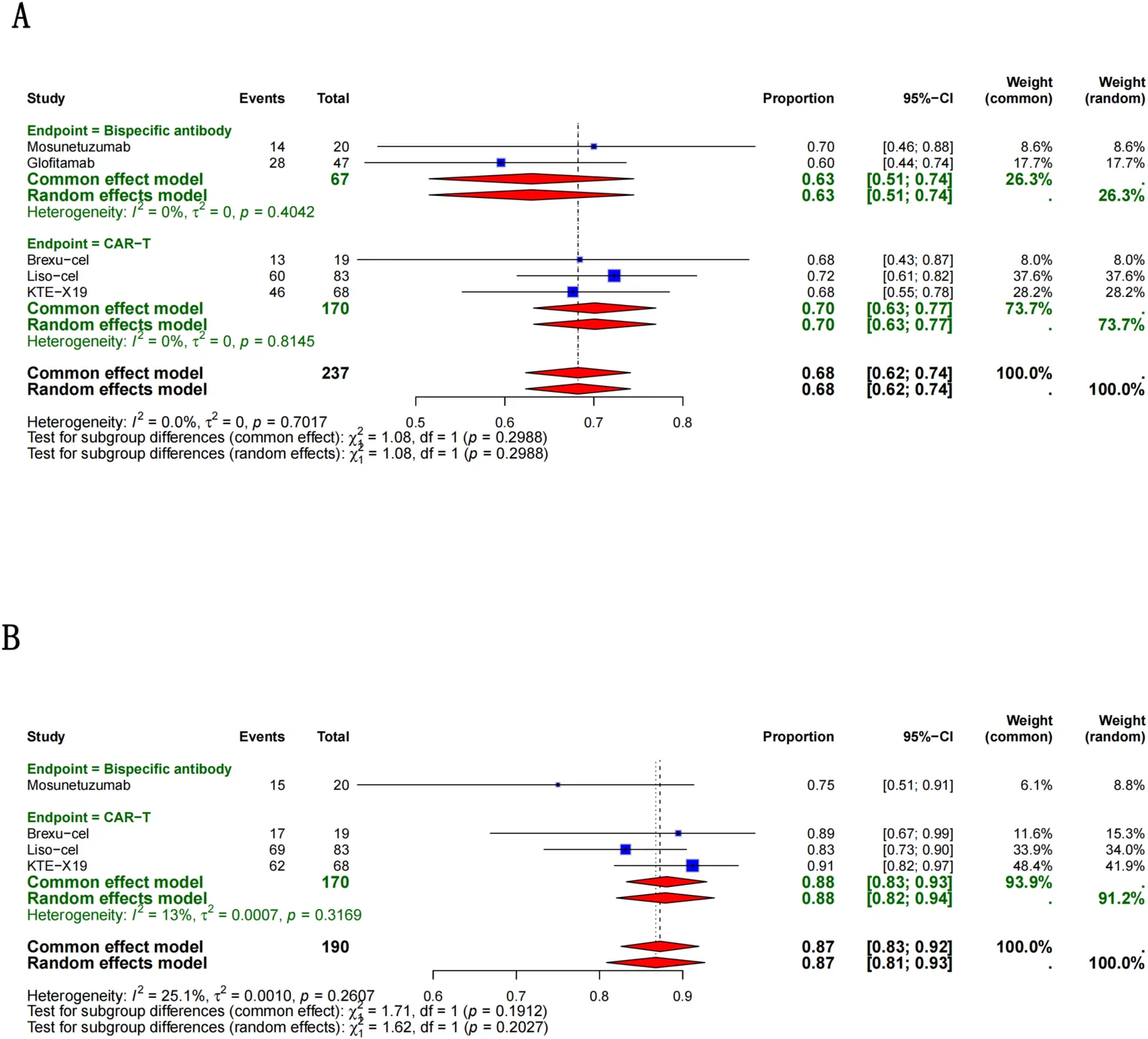
Forest plots comparing efficacy between bispecific antibodies and CAR-T therapies. **(A)** Complete Response (CR) rates: Forest plot comparing the CR rates between bispecific antibodies and CAR-T therapies. The pooled CR rates for bispecific antibodies were 0.63 (95% CI, 0.51–0.74), while for CAR-T therapies, they were 0.70 (95% CI, 0.63–0.77). **(B)** Overall Response Rates (ORR): Forest plot showing the pooled ORR for bispecific antibodies (0.75, 95% CI, 0.51–0.91) and CAR-T therapies (0.88, 95% CI, 0.82–0.94).

The overall pooled CR rate for all therapies was 0.68(95% CI, 0.62-0.74). When stratified by treatment type, the CR rate was 0.63(95% CI, 0.51-0.74) for bispecific antibodies and 0.70(95%CI, 0.63-0.77) for CAR T-cell therapies (P < 0.05) (**Figure 2A**). The overall pooled OR rate was 0.87 (95% CI, 0.81-0.93), with OR rates of 0.75(95% CI, 0.51-0.91) for bispecific antibodies and 0.88(95%CI, 0.81-0.93) for CAR T therapies(P<0.05) (**Figure 2B**).

### 3.4 Adverse events

**Figure 3:**
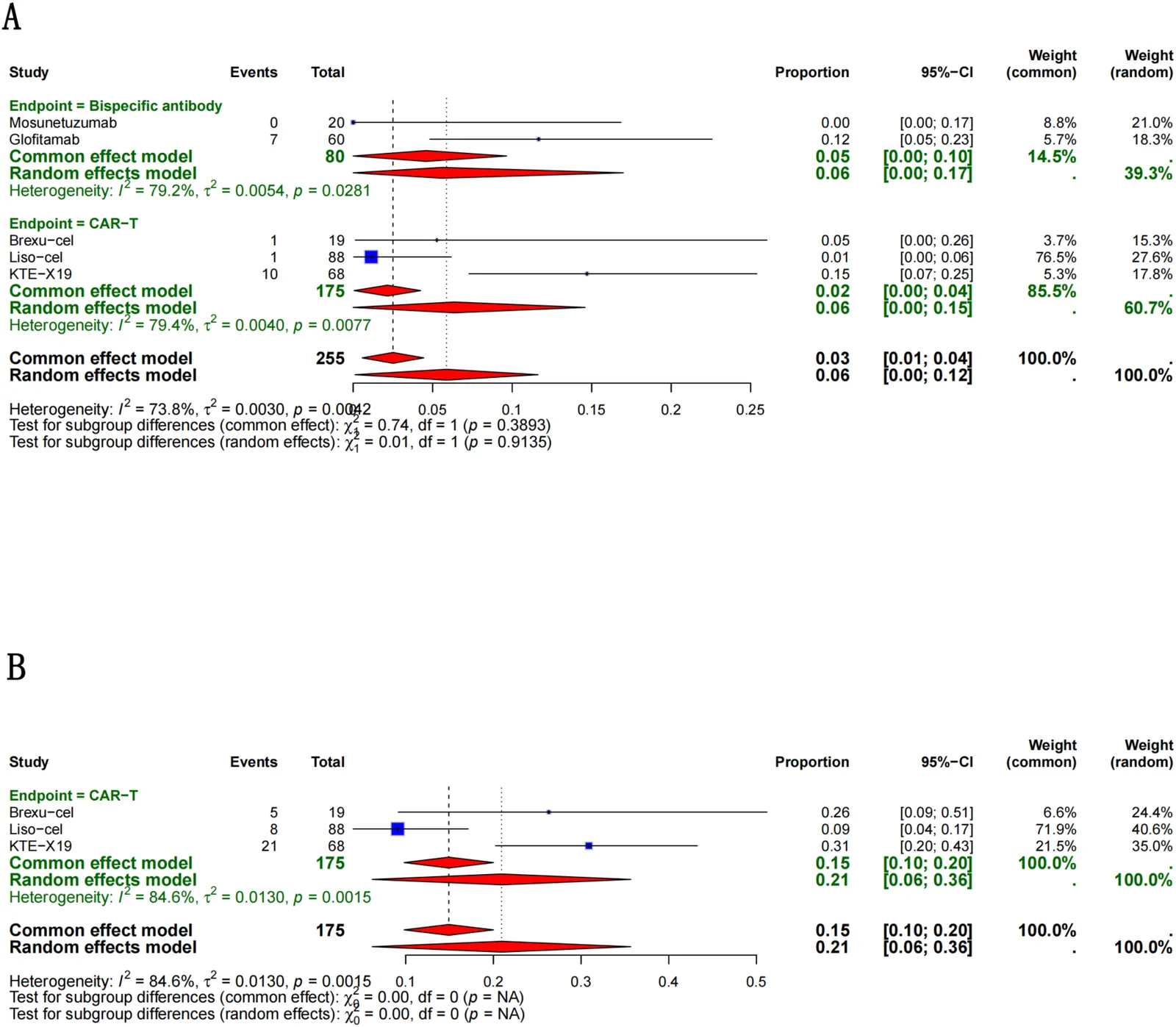
Forest plots analysis comparing grade 3 or higher adverse events of bispecific antibodies and CAR-T therapies. **(A)** ≥Grade 3 Cytokine Release Syndrome (CRS): Comparison of the incidence of ≥3 grade CRS: The incidence in the bispecific antibody group (0.06, 95% CI, 0.00 - 0.17) was comparable to that in the CAR-T therapy group (0.06, 95% CI, 0.00 - 0.15). **(B)** The incidence of grade 3 or higher neurological adverse events in the CAR-T treatment group was 0.21, with a 95% confidence interval of 0.06 to 0.36.

The pooled incidence of Grade ≥3 CRS was 0.06(95% CI, 0.00-0.17) for bispecific antibodies and 0.06(95%CI, 0.00-0.12) for CAR T-cell therapies(P > 0.05; **Figure 3A**). For neurological events of grade ≥ 3, one study in the bispecific antibody group did not provide relevant data. Another study had an incidence rate of 0, which was not comparable. Therefore, we analyzed separately the incidence rate of CAR T cell therapy in mantle cell lymphoma, which was 0.21 (95% CI, 0.06 - 0.36; **Figure 3B**).

### 3.5 Subgroup analysis

**Figure 4:**
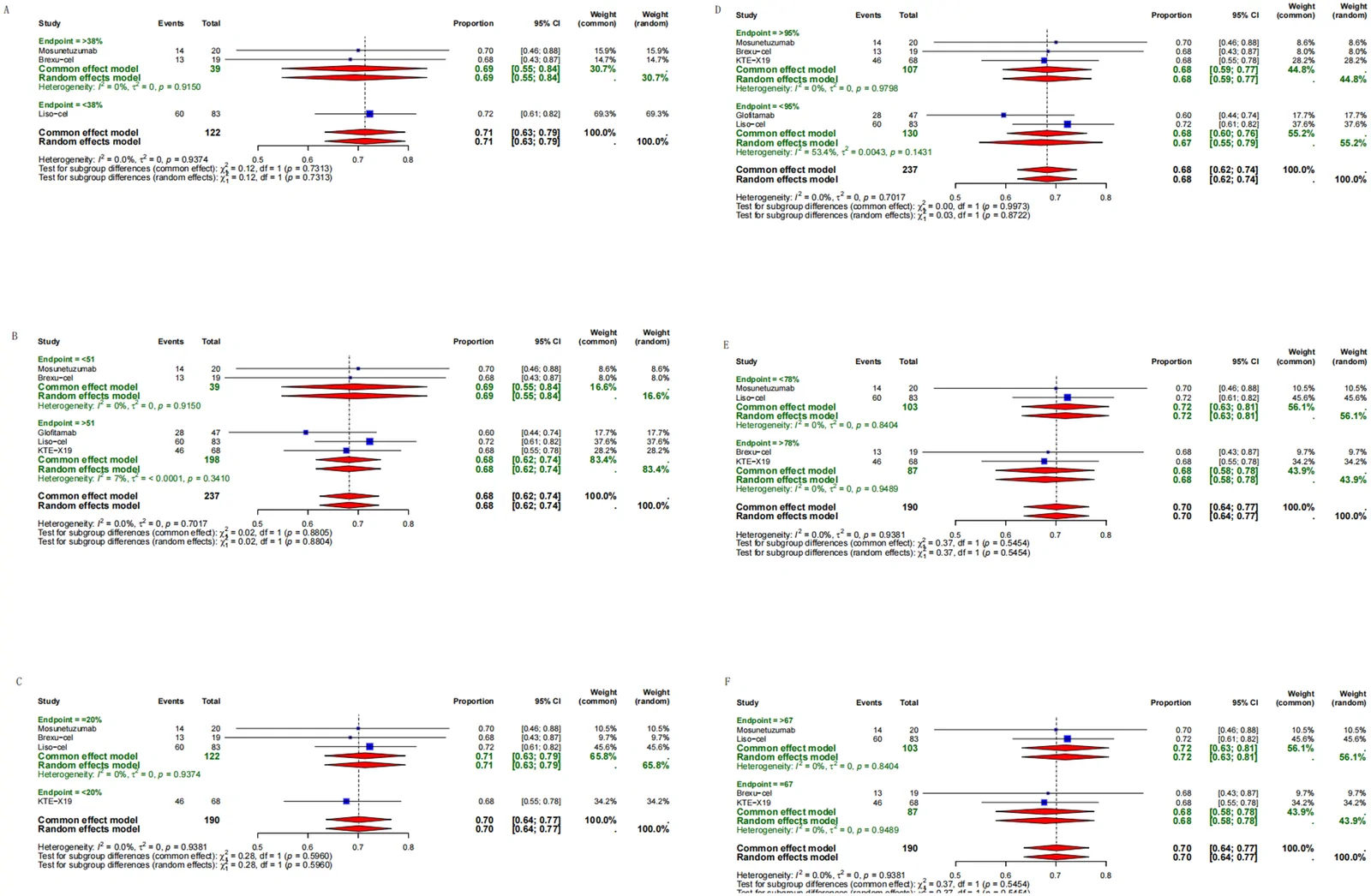
Forest plots of subgroup analyses for complete response rates based on study-level characteristics. **(A)** Stratified by proportion of patients with high-risk MIPI score (>38% vs. <38%); **(B)** stratified by study sample size (>51 vs. <51 patients); **(C)** stratified by proportion of patients with TP53 mutations (≥20% vs. <20%); **(D)** stratified by proportion of patients with prior BTKi therapy (>95% vs. <95%); **(E)** stratified by proportion of patients with Ki-67 ≥30% (>78% vs. <78%); **(F)** stratified by age (>67 years vs. ≤67 years).

To explore the potential impact of different baseline patient characteristics on treatment efficacy and to investigate sources of heterogeneity, we conducted subgroup analyses based on available study-level data (**Figure 4**). Subgroup cutoffs were determined using the median or mean proportions of patients with specific characteristics across the included studies.

In the subgroup stratified by the proportion of patients with high-risk MIPI score (>38% vs. <38%), the pooled CR rates were 0.69 (95% CI, 0.55–0.84) and 0.72 (95% CI, 0.61–0.82), respectively (P = 0.731; **Figure 4A**). When grouped by study size (>51 vs. <51), the pooled CR rates were 0.68 (95% CI, 0.62–0.74) and 0.69 (95% CI, 0.55–0.84), respectively (P = 0.880; **Figure 4B**). For the proportion of patients with TP53 mutations (≥20% vs. <20%), the pooled CR rates were 0.71 (95% CI, 0.63–0.79) and 0.68 (95% CI, 0.55–0.78), respectively (P = 0.596; **Figure 4C**). In the subgroup analysis based on the proportion of BTKi-treated patients (>95% vs. <95%), the pooled complete response rates were 0.68 (95% CI 0.59–0.77) for the >95% group and 0.67 (95% CI 0.55–0.79) for the <95% group, with no significant difference between subgroups (P = 0.872; **Figure 4D**).For the proportion of patients with Ki-67 ≥30% (>78% vs. <78%), the pooled CR rates were 0.68 (95% CI, 0.58–0.78) and 0.72 (95% CI, 0.63–0.81), respectively (P = 0.545; **Figure 4E**). Finally, in the age subgroup (>67 years vs. ≤67 years), the pooled CR rates were 0.72 (95% CI, 0.63–0.81) and 0.68 (95% CI, 0.58–0.78), respectively, with no statistically significant difference between subgroups (P = 0.545; **Figure 4F**).

Overall, none of the subgroup comparisons reached statistical significance (all interaction P > 0.05), suggesting that the CR benefit of CAR T-cell therapy over bispecific antibodies was generally consistent across these clinically relevant baseline characteristics.

### 3.6 Sensitivity analysis

**Figure 5:**
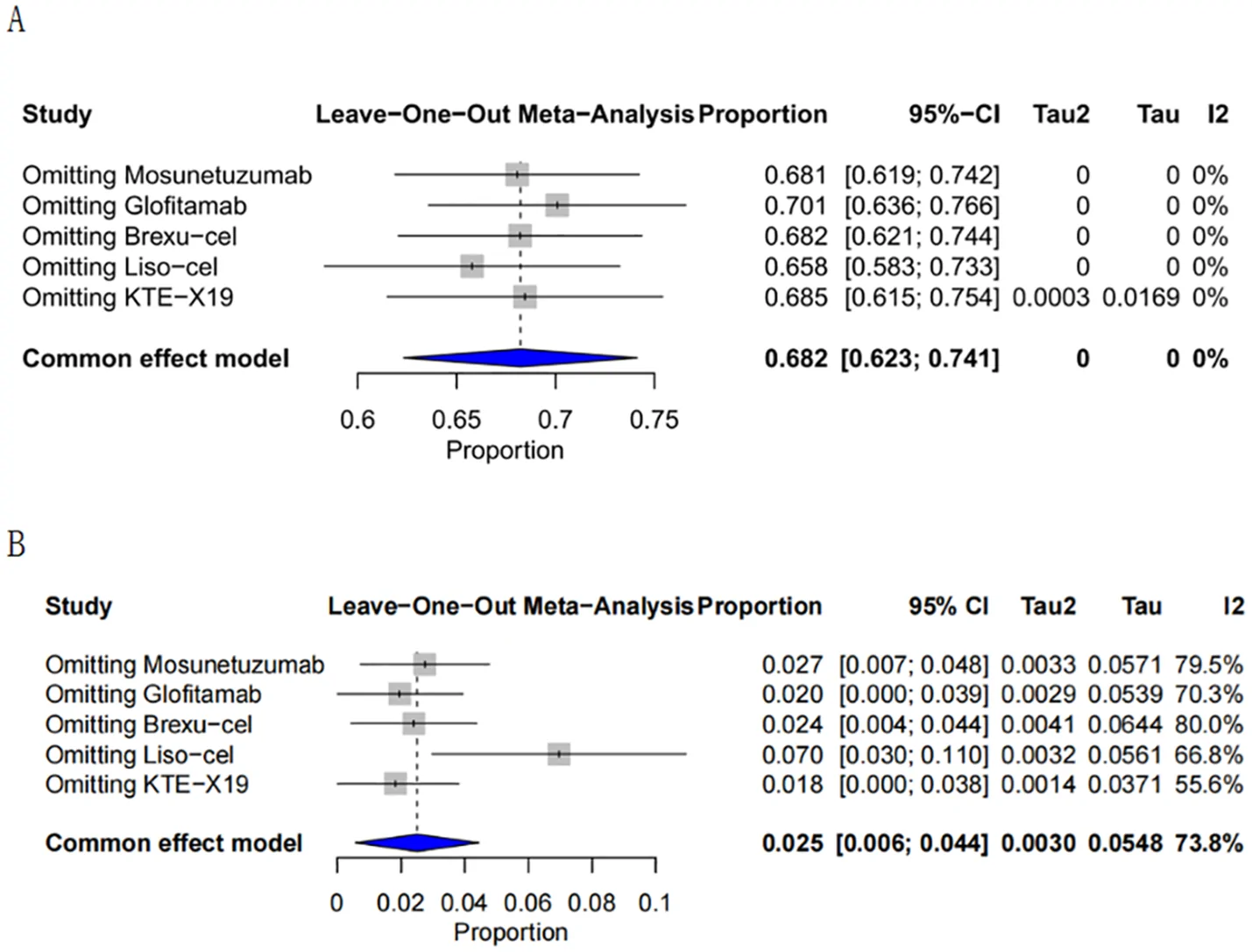
The sensitivity analysis of this meta-analysis. **(A)** Leave-one-out sensitivity analysis for complete response (CR) rates. **(B)** Leave-one-out sensitivity analysis for grade ≥3 cytokine release syndrome (CRS).

Sensitivity analyses confirmed the robustness of the results. When using the fixed effects model to test the results of the random effects model, we can observe that the efficacy difference between the two treatment methods still exists (**Figure 2A**). Additionally, when using the leave-one-out analysis, we found that after excluding any single study, the range of changes in the combined proportion was relatively small, ranging from 0.658 to 0.701. The maximum change occurred after excluding Liso-cel (from 0.682 to 0.658), with a relative change of approximately 3.5% and overlapping confidence intervals. The 95% confidence intervals of all sensitivity analysis results all overlapped with the confidence intervals of the original analysis. Moreover, in all analyses, after excluding any study, the consistency of I^2^ remained unchanged and was at a low level, indicating that the heterogeneity among the studies was small (**Figure 5A**).

To further assess the robustness of the safety findings, we also performed a leave-one-out sensitivity analysis for the incidence of grade ≥3 cytokine release syndrome (CRS). After sequentially excluding each individual study, the pooled incidence of grade ≥3 CRS ranged from 0.018 to 0.070, and the 95% confidence intervals of all estimates overlapped with that of the original pooled estimate. The I^2^ values ranged from 55.6% to 80.0% across the leave-one-out analyses; no single study exclusion reduced the I^2^ below 50%, suggesting that the observed moderate-to-high heterogeneity in the safety analysis was likely attributable to systematic differences across multiple studies (e.g., variations in CRS grading criteria or management protocols) rather than being driven by any single study(**Figure 5B**). Collectively, these sensitivity analyses support the robustness of both the primary efficacy estimate and the safety estimate for grade ≥3 CRS, while acknowledging that heterogeneity in safety outcomes warrants cautious interpretation.

## 4 Discussion

This meta-analysis provides a systematic comparison of CAR T-cell therapy and CD20×CD3 bispecific antibodies in the treatment of relapsed/refractory mantle cell lymphoma (R/R MCL). Both therapies have shown remarkable potential in improving outcomes for heavily pretreated patients; however, their efficacy and safety profiles exhibit significant differences, which can guide clinical decision-making.

The pooled complete response (CR) rate was significantly higher for CAR T-cell therapy (0.70,95%CI,0.63-0.77)) compared to bispecific antibodies (0.63,95% CI,0.51-0.74)) (P < 0.05). This suggests that CAR T-cell therapy may achieve superior tumor control in certain patient populations. The overall response rates of the two therapies (CAR T therapies: 0.88, 95% CI, 0.81 - 0.93; Bispecific antibodies: 0.75, 95% CI, 0.51 - 0.91) also support the above results. CAR T cell therapy can offer personalized and long-lasting immune responses, while bispecific antibodies, as a “ready-made” treatment option, also possess certain pharmacodynamic properties.

In terms of safety, the incidence of grade ≥ 3 CRS was comparable between the two therapies (CAR T-cell therapies: 0.06, 95% CI, 0.00 - 0.12; Bispecific antibodies: 0.06, 95% CI, 0.00 - 0.17, P > 0.05). This indicates that both treatment regimens share a common risk of T-cell activation. However, it is important to note that the management strategies for CRS, including the use of prophylactic tocilizumab, were not uniform across the included studies. While step-up dosing was a common strategy for bispecific antibodies to mitigate CRS, prophylactic tocilizumab was generally not mandated in the pivotal CAR T-cell trials, relying instead on institutional protocols for Grade ≥2 CRS. This heterogeneity in supportive care underscores the need for standardized guidelines to improve safety management. Regarding the incidence of ≥3 grade ICANS, due to the limited data volume in the bispecific antibody group, no comparison could be made. However, based on our data analysis, the incidence of ≥3 grade ICANS in the CAR T-cell therapy group was 0.21 (95% CI, 0.06 - 0.36).These findings underline the need for improved management strategies to mitigate the risks of CRS and neurotoxicity, especially for CAR T-cell therapy, which requires intensive monitoring and specialized care during administration.

To explore potential sources of heterogeneity and to assess whether the superior CR rate of CAR T-cell therapy was consistent across different patient and study characteristics, we performed multiple subgroup analyses based on study-level data. The results showed that none of the evaluated factors—including age (>67 vs. ≤67), study size (>51 vs. ≤51), proportion of high-risk MIPI (>38% vs. <38%), prior BTKi exposure (>95% vs. <95%), proportion of patients with Ki-67 ≥30% (>78% vs. ≤78%), or TP53 mutation status (≥20% vs. <20%)—significantly modified the relative efficacy difference between the two therapies (all interaction P > 0.05). Within-group heterogeneity was low (I^2^ = 0%) in most subgroups, with moderate heterogeneity (I^2^ = 53.4%) detected only in the subgroup with a lower proportion of BTKi-treated patients (≤95%), which may be attributable to differences in patient populations, prior treatment lines, or drug-specific characteristics (e.g., glofitamab vs. liso-cel) across the included studies. Collectively, these subgroup findings support the robustness of the primary efficacy estimate, suggesting that the CR advantage of CAR T-cell therapy remains broadly consistent across clinically relevant subgroups. However, given that some subgroups contained only 1–2 studies, these results should be interpreted as exploratory.

Furthermore, resistance mechanisms are a major and growing concern in T-cell-engaging therapies. Resistance mechanisms to CAR T-cell therapy are multifactorial. Mouhssine and colleagues systematically reviewed the molecular determinants of resistance to CAR T-cell therapy in large B-cell lymphoma, categorizing resistance mechanisms into tumor-intrinsic factors, CAR T-cell-related factors, and tumor microenvironment (TME) factors (14). Tumor-intrinsic factors primarily include downregulation or loss of target antigen (e.g., CD19) expression, along with genomic alterations such as CD19 mutations and chromothripsis (15–17); CAR T-cell-related factors encompass T-cell exhaustion, suboptimal differentiation phenotypes, insufficient expansion, and limited *in-vivo* persistence (18, 19); while immunosuppressive cells, metabolic constraints, and hypoxia within the TME further restrict CAR T-cell infiltration and function (20, 21). Resistance mechanisms to bispecific antibodies are similarly complex. Studies have shown that bispecific antibody resistance is also multifactorial, involving tumor-related factors (such as CD20 downregulation, MS4A1 mutations, and genomic alterations including TP53, MYC, and NOTCH1), T-cell characteristics (including inadequate intratumoral CD8+ T-cell infiltration and T-cell exhaustion marked by upregulation of PD-1, LAG-3, and TIM-3), and the immunosuppressive TME (22). Antigen escape also represents a critical resistance mechanism for bispecific antibodies. Differences in resistance between the two therapies warrant attention. Both face common challenges including antigen escape, T-cell dysfunction, and an immunosuppressive microenvironment. However, as a “living drug,” CAR T-cell therapy resistance more frequently involves cell-intrinsic factors such as CAR T-cell persistence, expansion capacity, and memory phenotype differentiation (23); whereas bispecific antibodies, as off-the-shelf agents, are more closely associated with endogenous T-cell functional reserve and exhaustion progression under chronic stimulation (24). We advocate for future systematic reviews and translational studies that directly compare these resistance mechanisms between the two modalities.

Despite its strengths, this meta-analysis has several limitations. First and foremost, the included studies were predominantly single-arm trials with a limited total sample size (only 237 patients across five studies). Consequently, our findings should be interpreted as hypothesis-generating rather than definitive. They provide a rationale for future research but are insufficient to guide a paradigm shift in clinical practice without confirmation from large, prospective, head-to-head trials. Second, while the median age was comparable across studies (65-68.5 years), the lack of individual patient data prevented us from performing subgroup analyses based on age, performance status, or comorbidities. This is a notable limitation, as these factors significantly influence treatment selection and tolerability. Third, the primary endpoint of this meta-analysis was the CR rate. While a higher CR rate is a clinically relevant endpoint, the absence of robust progression-free survival (PFS) and overall survival (OS) data in several included studies is a significant limitation. The comparison of durability of responses between CAR T-cell therapy and bispecific antibodies remains a major knowledge gap. Fourth, the comparison of safety profiles, particularly for neurotoxicity, was hampered by considerable statistical heterogeneity and incomplete reporting. We acknowledge that the high I^2^ values (≈80%) suggest substantial variability among studies in toxicity grading and management, which limits the reliability of the pooled safety estimates. Finally, data on infectious complications, an important safety endpoint, were not uniformly reported, preventing a meaningful comparative analysis. We emphasize the importance of standardized and detailed reporting in future trials.

Despite these limitations, our findings offer valuable clinical insights. The advantage of CAR T-cell therapy in depth of remission must be weighed against its higher cost, complex manufacturing process, and potential for severe neurotoxicity. Bispecific antibodies, as an off-the-shelf treatment, offer distinct advantages in accessibility and convenience, with a more predictable and manageable CRS profile. Treatment selection should consider patient baseline status, disease aggressiveness, therapeutic goals (deep remission vs. disease control), and healthcare resource availability. This study provides an indirect comparison based on single-arm trials and should not be interpreted as a head-to-head comparative effectiveness analysis. Future large-scale, prospective, head-to-head randomized controlled trials, complemented by individual patient data meta-analyses, are urgently needed to further validate the comparative efficacy and safety of these two therapies. In parallel, biomarker-driven stratification strategies and in-depth investigation of resistance mechanisms will facilitate the identification of optimal patient populations for each approach, advancing the precision treatment of R/R MCL.

In summary, this meta-analysis highlights the unique advantages of CAR T cell therapy, especially in the treatment of relapsed/refractory mantle cell lymphoma (R/R MCL). Compared with bispecific antibody therapy, it can achieve a higher complete response rate (CR), but the ≥3 grade adverse events (CRS and ICANS) require attention. However, both therapies remain viable options, and their use should be tailored to individual patient characteristics, disease biology, and resource availability. Future head-to-head RCTs and long-term follow-up studies are needed to further elucidate the comparative efficacy and safety of these promising immunotherapeutic strategies.

## Data Availability

The original contributions presented in the study are included in the article/supplementary material. Further inquiries can be directed to the corresponding authors.
